# Intervertebral disc degeneration in warmblood horses: Histological and biochemical characterization

**DOI:** 10.1177/03009858211067463

**Published:** 2022-01-04

**Authors:** Wilhelmina Bergmann, Chris van de Lest, Saskia Plomp, Johannes C. M. Vernooij, Inge D. Wijnberg, Willem Back, Andrea Gröne, Mark W. Delany, Nermin Caliskan, Marianna A. Tryfonidou, Guy C. M. Grinwis

**Affiliations:** 1 Department of Biomolecular Health Sciences, Faculty of Veterinary Medicine, Utrecht University, Utrecht, The Netherlands; 2 Department of Clinical Sciences, Faculty of Veterinary Medicine, Utrecht University, Utrecht, The Netherlands; 3 Department of Population Health Sciences, Faculty of Veterinary Medicine, Utrecht University, Utrecht, The Netherlands; 4 Department of Surgery and Anaesthesia of Domestic Animals, Faculty of Veterinary Medicine, Ghent University, Merelbeke, Belgium; * Current address: Diergezondheidszorg Vlaanderen (DGZ), Torhout, Belgium.

**Keywords:** fibrosis, glycosaminoglycan, scoring, histology, horse, hydroxylysine, intervertebral disc degeneration, pentosidine

## Abstract

Gross morphology of healthy and degenerated intervertebral discs (IVDs) is largely similar in horses as in dogs and humans. For further comparison, the biochemical composition and the histological and biochemical changes with age and degeneration were analyzed in 41 warmblood horses. From 33 horses, 139 discs and 2 fetal vertebral columns were evaluated and scored histologically. From 13 horses, 73 IVDs were assessed for hydration, DNA, glycosaminoglycans, total collagen, hydroxyl-lysyl-pyridinoline, hydroxylysine, and advanced glycation end-product (AGE) content. From 7 horses, 20 discs were assessed for aggrecan, fibronectin, and collagen type 1 and 2 content. Histologically, tearing of the nucleus pulposus (NP) and cervical annulus fibrosus (AF), and total histological score (tearing and vascular proliferation of the AF, and chondroid metaplasia, chondrocyte-like cell proliferation, presence of notochordal cells, matrix staining, and tearing of the NP) correlated with gross degeneration. Notochordal cells were not seen in IVDs of horses. Age and gross degeneration were positively correlated with AGEs and a fibrotic phenotype, explaining gross degenerative changes. In contrast to dogs and humans, there was no consistent difference in glycosaminoglycan content and hydration between AF and NP, nor decrease of these variables with age or degeneration. Hydroxylysine decrease and collagen 1 and AGEs increase were most prominent in the NP, suggesting degeneration started in the AP. In caudal cervical NPs, AGE deposition was significantly increased in grossly normal IVDs and total collagen significantly increased with age, suggesting increased biomechanical stress and likelihood for spinal disease in this part of the vertebral column.

Neurological signs originating from the neck are common in the horse.^62^ The most common noninfectious, nontraumatic cause is cervical vertebral myelopathy.^33,37^ In this syndrome, neurological signs are caused by compression of the spinal cord and spinal nerves in which vertebral instability and enlargement of the articular process joints and thickening of the vertebral dorsal laminae and ligamentum flavum are important factors.^16,35,41^ However, a link between cervical vertebral myelopathy and intervertebral disc degeneration (IVDD) has not been definitively established. This study aims to assess IVDD in horses, as a basis for considering its possible role in cervical vertebral myelopathy in horses.

Cervical neurological signs due to compression of nervous tissue have also been commonly described in dogs and humans, and in these species IVDD and subsequent disc disease is one of the main underlying lesions.^6,30^ Degeneration of the canine and human intervertebral disc (IVD) is characterized by changes of the cellular composition and the extracellular matrix of the nucleus pulposus (NP), annulus fibrosus (AF), transition zone, and end plate.^7,9^ On a cellular level, disc maturation in humans and degeneration in dogs and humans is characterized by replacement of physaliferous notochordal cells in the NP and fibroblasts in the AF by smaller, nonvacuolated chondrocyte-like cells with corresponding matrix deposition^12,26^ (chondroid metaplasia), and in the AF by subsequent disorganization of the collagenous lamellae.^7,9,12,46^

In both canine and human NPs, the most characteristic biochemical finding associated with degeneration is loss of proteoglycans and their attached sulfated glycosaminoglycans (GAGs) with subsequent dehydration of the NP.^1,2,8^ A second important biochemical change seen in dogs and humans is the development of a fibrotic matrix, which is characterized by an increase in the total amount of collagen in the NP and the AF^1,9^ and a decrease in the amount of type 2 collagen in the NP with a simultaneous increase of type 1 collagen.^4,7,9,66^ Furthermore, fibronectin, which is implicated in fibrosis,^57^ increases in the degenerated NP in dogs and in the degenerated AF and NP in humans.^17,38,66^

Collagen cross-links, such as hydroxyl-lysyl-pyridinoline (HP), are vital for the biomechanical properties of the extracellular matrix including that of the IVD.^20,29,63^ Hydroxylysine is important for the formation of these cross-links and is formed posttranslationally by hydroxylation of the amino acid lysine.^14^ Furthermore, hydroxylysine may undergo posttranslational enzymatic O-glycosylation. Although the exact function of these attachments of sugar groups is yet unknown, recent literature suggests that the extent and pattern of glycosylation of hydroxylysine may regulate cross-link maturation in collagen and therefore modulate its properties.^14,53,56^

In equine and in human IVDD, yellow discoloration of the disc is a prominent gross finding.^1,10^ A likely cause for this change in color is increased nonenzymatic glycation of proteins leading to the formation of advanced glycation end-products (AGEs). AGEs form abnormal collagen cross-links resulting in structural tissue modifications with subsequent changes in the biomechanical properties of the disc.^1,3,20,63^

Ultimately, these cellular and biochemical changes can lead to structural and functional failure of the IVD, resulting in subsequent failure to limit spinal movement and an inability to resist compression. This can further affect other parts of the spinal unit such as the articular process joints.^1,4,9^

Cervical IVDD is common in warmblood horses and grossly comparable to dogs and humans.^10^ Herniation or protrusion of the IVD are considered rare in horses,^11,52^ and systematic studies on the relation with clinical signs are missing. Clinical signs due to functional failure of the degenerated IVD and subsequent pathological changes of other parts of the spinal unit seem likely and might be underreported or underdiagnosed.

The overall objectives of this study were to investigate the association of cellular and biochemical changes of the equine IVD with age and degeneration. For this, a histological scoring scheme for degenerative changes was developed in which also the presence of notochordal cells was included. Furthermore, the biochemical changes associated with age and IVDD in dogs and humans (changes in hydration, GAG, aggrecan, and collagen content) were examined in horses. Additionally, changes of the quality of collagen with age and degeneration were investigated by evaluation of hydroxylysine and HP content and degree of nonenzymatic glycation by quantifying the AGE biomarker pentosidine.^47,48^ It was hypothesized that the cellular and biochemical changes in equine IVD are similar to those of dogs and humans.

## Materials and Methods

### Horses

Vertebral columns of 41 warmblood horses, including 2 fetuses (approximately 45 days and 61 days of gestation^24^), were harvested postmortem (Supplemental Table S1). Of these, 38 privately owned horses were referred for necropsy, with the owner’s informed consent, to the Division of Pathology, Faculty of Veterinary Medicine, Utrecht University. These horses had either died unexpectedly or were humanly euthanized for reasons unrelated to the current study. The fetal IVDs were derived from a pregnant mare undergoing necropsy and from an aborted fetus from an unrelated reproduction study (approved by the university’s Animal Experimentation Committee [permission number 2007.III.02.036]). Finally, one experimental horse was included, which was owned by the Division of Equine Sciences of Utrecht University and used for teaching purposes, which included obtaining surgical skills with subsequent euthanasia (permission number 2013.III.01.012).

The age ranged from approximately 45 days gestation up to 21 years with a mean age of 7.0 ± 7.4 years. The horses were of different breeds (38 Royal Dutch Sport horses, 2 Zangersheide horses, 1 Holsteiner) and sex (19 mares, 8 stallions, 11 geldings, and 3 of unknown sex). The IVDs of horses of 8 months and older (*N* = 26), were examined in a previous study concerning macroscopic grading and distribution of equine IVDD along 4 different regions of the spine.^10^ These regions were the cranial cervical region from cervical vertebrae (C)2–C5, the caudal cervical region from C5 to thoracic (T)1, T11–T13, and the lumbosacral region L4–S1.

To meet the desired minimum number of 10 IVDs per gross grade for biochemical evaluation, 2 T1–T2 IVDs with gross grade 5 were also collected. These vertebral regions were dissected, sectioned mid-sagittally, photographed, and macroscopically graded as described.^10^ In this previously developed 5-point grading scheme, grades 1 and 2 were considered normal, grade 3 a transitional grade, and grades 4 and 5 severely degenerated.

### Histological Preparation

Of a subgroup of 33 horses (Supplemental Table S1), 139 IVDs and 2 complete fetal vertebral columns were evaluated histologically. IVDs containing the NP, AF, cartilaginous endplate, and subchondral bone of 9 months gestation and older horses were cut into 0.5-cm-thick slices using a band saw, fixed in 10% (v/v) neutral buffered formalin, and decalcified for about 9 months in 10% (w/v) ethylenediaminetetraacetic acid with a pH of 7, before routine processing and staining with hematoxylin/eosin and with Alcian blue/picrosirius red for optimal detection of both collagen and proteoglycan-containing matrix components in the same histology section.^23^ In the fetuses, the entire vertebral columns were cut sagittally with a microtome knife, fixed in 10% neutral-buffered formalin, routinely processed, and stained with hematoxylin/eosin and Alcian blue/picrosirius red.

### Validation of the Histologic Scoring Scheme

After initial histological evaluation of the IVDs, it was strongly suspected that certain histological differences were age-dependent, and a provisional histologic scoring scheme for equine IVDD was devised (Supplemental Table S2) that was largely based on the validated scoring scheme for the dog.^7^ For validation of this histological scoring scheme, 10 IVDs with each macroscopic grade for degeneration (1 to 5) were selected. This total of 50 IVDs were assigned a random number using an online randomization program and subsequently scored blindly twice by 3 independent observers with a time lapse of at least a week between scoring sessions for each observer.

### Notochordal Cells

To evaluate the presence of notochordal cells, the vertebral columns of the 2 fetuses and 1 adult horse (11 years old) were immunolabeled for cytokeratin 18.^45^ The positive controls were an IVD of a non-chondrodystrophic dog with notochordal cells in the NP, and kidneys of an adult horse and the fetus of 45 days gestation.^43,45^ After deparaffinization and rehydration of the sections, antigen retrieval was achieved by incubating with citrate buffer (Dako Target Retrieval Solution, Dako) of pH 6 at 97 °C with Dako PT-link (Dako) for 20 minutes. To prevent nonspecific binding, peroxidase blocking solution (Dako) and 10% normal goat serum were added for 5 minutes and 15 minutes, respectively, at room temperature (RT). Thereafter, the slides were incubated with the primary antibody for cytokeratin 18 (0.5 µg/mL, monoclonal, goat anti-mouse, Abcam [C-04] ab 668, lot: GR3196069-6) in normal antibody diluent (Bright Diluent Green, VWR; 60 minutes, RT). Then, the sections were incubated with the secondary antibody (Brightvision Goat Anti-Mouse/Rabbit, VWR; 30 minutes, RT), and subsequently with AEC (Agilent; 20 minutes, RT). Nuclei were counterstained with hematoxylin for 20 seconds at RT.

### Biochemical Analysis of the Equine IVD

Biochemical analyses were performed on a subgroup of 13 horses (Supplemental Table S1) with a total of 73 IVDs that were collected within 24 hours after death, including 22 IVDs with grade 1 for gross degeneration, 30 with grade 2, 11 with grade 3, 1 with grade 4, and 9 with grade 5. The IVDs with grades 4 and 5 were combined to form a group of 10 severely degenerated discs. The distribution of the grades along the different examined spinal regions is shown in Supplemental Table S3. IVDs with grades 4 and 5 for gross degeneration were only found in the caudal cervical and cranial thoracic regions (C5–T2). These 2 regions did not contain any IVDs with grade 3 for gross degeneration. Biochemical evaluation was done for all examined IVDs combined (all IVDs from all researched vertebral regions), but also for the different regions separately, except for region T1–T2 from which only 2 IVDs were sampled.

**Figure 1.**
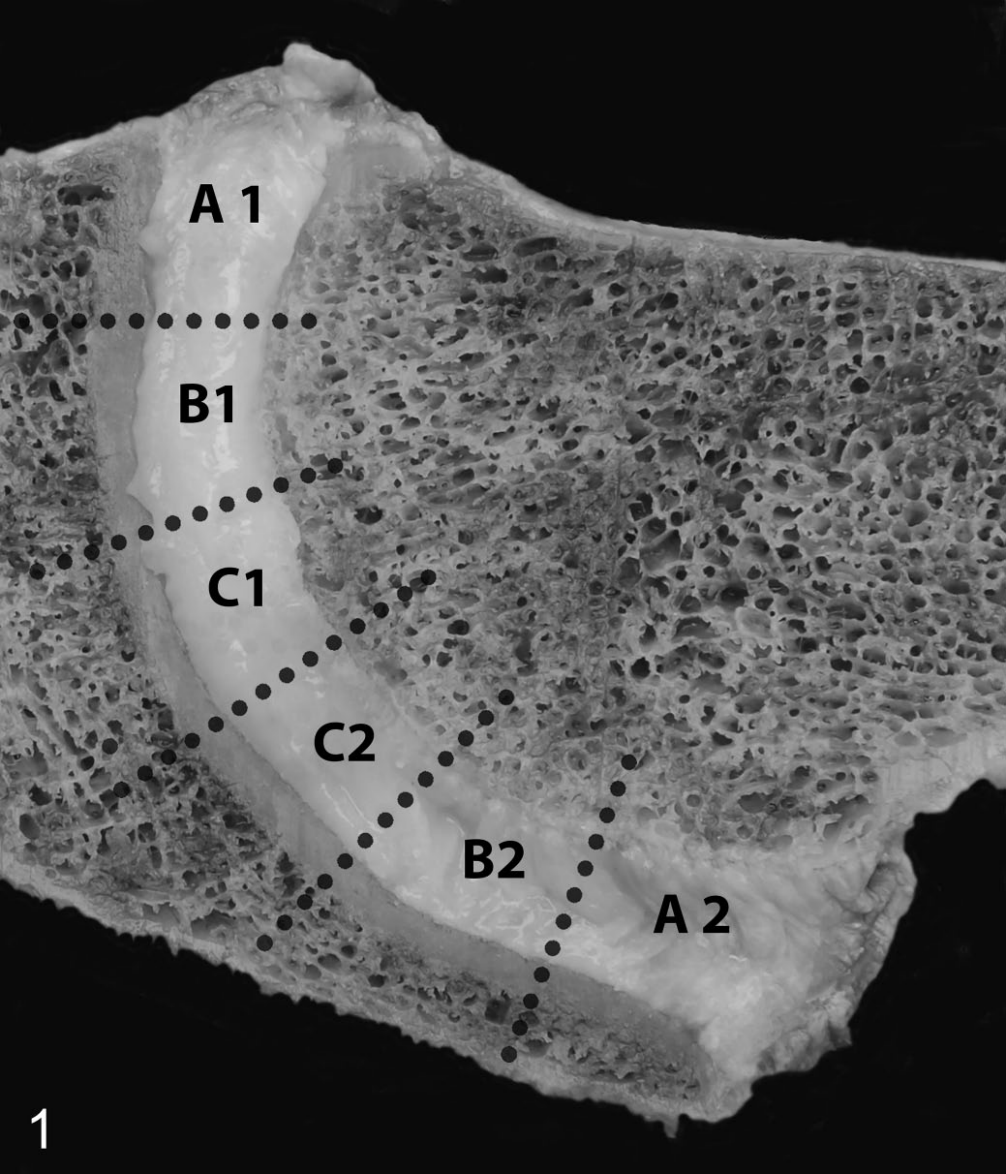
Normal intervertebral disc (IVD), C3–C4, horse. Mid-sagittal vertebral section. For biochemical evaluation, IVDs were cut into 6 equally large pieces representing the annulus fibrosus (areas A1 and A2), the nucleus pulposus (areas C1 and C2), and the transition zone (areas B1 and B2).

Within 24 hours after death, IVDs were photographed during necropsy and a sagittal full-thickness, 0.5-cm-thick section was prepared including the AF, transition zone, and the NP. This section was cut into 6 equally sized pieces representing the AF (areas A1 and A2), the NP (areas C1 and C2), and the transitional zone between AF and NP (areas B1 and B2; Fig. 1). Subsequently, these pieces of tissue were snap frozen in liquid nitrogen and stored at −80 °C until further analysis.

Each of the samples of the IVD were further randomly cut into 4 equal portions, put into microcentrifuge safe-lock tubes (Eppendorf), and wet weights were measured with a high-resolution analytical balance scale (Mettler Toledo AB). Dry weights were determined after overnight lyophilization by using a SpeedVac Vacuum Concentrator (Thermo-Fisher). Two of the 4 portions of each sample were used for further biochemical analysis. Because there was no statistically significant difference in hydration between the AF and NP, nor a statistically significant correlation with hydration and age or degeneration, the DNA, GAG, and total collagen content are expressed in milligrams (DNA, GAG) or grams (total collagen) per gram wet weight.

Two samples of AF and NP per IVD were digested with papain as described before.^59^ To determine the cellularity of the tissue, DNA content was determined by fluorometric quantitation with a Quant-iT dsDNA Broad-Range assay kit in combination with Qubit (Invitrogen) according to the manufacturer’s protocol. Subsequently, a modified 1.9-dimethylmethylene blue assay was performed to establish the GAG content.^19^ Both DNA and GAG content are expressed as mg/g wet weight.

After papain digestion, 2 samples of AF and NP per IVD were hydrolyzed in a 6 M hydrochloric acid solution in milliQ water (Millipore). Subsequently, the hydroxyproline (to determine the collagen content), hydroxylysine, HP, and pentosidine contents were quantified by high-performance liquid chromatography mass spectrometry (HPLC/MS) as described previously.^51^ Only samples with a minimal recovery of 30% were included in further analysis. Total collagen content is expressed as g/g wet weight. Hydroxylysine is expressed as percentage of total lysine. HP and pentosidine are expressed as mole per mole of collagen.

Of the IVDs used for biochemical analysis, 20 cervical IVDs of 7 horses (mean age 16 ± 1 years, all Royal Dutch Sport horses, 4 mares, 3 geldings) were used to determine the difference in the amount of aggrecan, collagen type 1α1 and type 1α2, collagen type 2α1, and fibronectin between normal IVDs (1 IVD with grade 1, 9 IVDs with grade 2) and severely degenerated discs (1 IVD with grade 4, 9 IVDs with grade 5). From these samples, 40-µm cryosections were collected on glass slides and dried. The tissue sections were washed twice in 0.1 M HCl and further processed as described.^28^ The identified peptides are expressed as percentage of the total signal.

### Statistical Analysis

To validate the histological scoring system, pairwise interobserver and intraobserver reliabilities were calculated for each individual variable with a Cohen’s weighted κ analysis with quadratic weights of 1, 0.9375, 0.75, 0.4375, and 0. A κ-value of less than 0.00 is considered poor, a value between 0.00 and 0.20 as slight, between 0.21 and 0.40 as fair, between 0.41 and 0.60 as moderate, between 0.61 and 0.80 as substantial, and a value between 0.81 and 1.00 as almost perfect.^32^ Also, the percentages of intraobserver and interobserver agreement per variable were calculated.

If the reliability of the histological scoring was considered sufficient (weighted κ values of fair or higher or observer agreement percentages of ≥80), the correlation between the individual histological variable and the macroscopic grade (1 to 5) and normal (grades 1 and 2) versus severely degenerated (grades 4 and 5) was determined. For this the 3 different scores obtained during the second round of scoring were averaged and a Pearson correlation test was performed. The strength of the correlation *r* was considered very weak (0.00–0.19), weak (0.20–0.39), moderate (0.40–0.59), strong (0.60–0.79), or very strong (0.80–1.00).^18^ Correlation of the total histological score of an IVD (determined by the sum of the averaged scores of those variables that could be scored reliably) with degeneration was also determined. Calculations were also performed for the cervical IVDs only (*n* = 32) because IVDD in this part of the vertebral column is most prevalent.^10^ Statistical significance was set at *P* ≤ .05. Analysis was conducted with SPSS 16.0 (IBM).

The results of the collagen, aggrecan, and fibronectin analyses were further processed using LCMS^2^ proteomics data analyses in R version 3.4.3 using the package MSGF plus version 1.19.1. The data of all biochemical variables, except for hydration, were not normally distributed. Due to the large number of variables, the initial use of a linear mixed model, which would take out horse-dependent variation, was not considered executable. Instead, in order to enable descriptive analysis of the data it was decided to first evaluate the composition of the IVDs and correlation of the variables with age and with gross degeneration by using heat maps of Spearman rank correlation coefficients. Heat maps were created for the IVDs of all regions combined, and for IVDs of the cranial cervical (C2–C5), caudal cervical (C5–T1), thoracic (T11–T13), and lumbosacral (L4–S1) regions separately. Furthermore, heat maps were created with only the data of normal discs (to determine the difference in composition of the AF and the NP, and to determine age-related changes), and the results of all grades (to determine degeneration-related differences). Also, separate heat maps were created for the AF and NP separately. Significance was set at *P* < .05. Interpretation of the strength of the correlation was the same as for the Pearson correlation coefficients.^18^

Furthermore, 2 linear mixed models were conducted to further investigate the formation of AGEs in the different spinal regions and the caudal cervical region more specifically. Therefore, one mixed model was used to determine regional differences in the initial formation of AGEs that may explain the higher prevalence of IVDD in the caudal cervical spine.^10^ For this purpose, the pentosidine content of the normal IVDs (grade 1 and grade 2) in the different regions was examined. By use of the second linear mixed model the pentosidine content in the different grades within this caudal cervical region were assessed. For this, the results of the pentosidine analyses were log-transformed to meet the model assumptions for normality and constant variance. In both models, “horse” was set as random effect and “region” and respectively “grade” as factor variables. Bonferroni correction was used to correct for multiple comparisons. To check for the correctness of the model, residuals were used. All linear mixed model calculations were done for the AF and NP separately. Heat maps and subsequent mixed models were performed in R version 3.4.3 using the corrplot and nlme packages.^39,55^ The raw data analyzed in this study are available in Supplemental Tables S4 and S5.

## Results

### Histological Changes With Age

**Figures 2–5.**
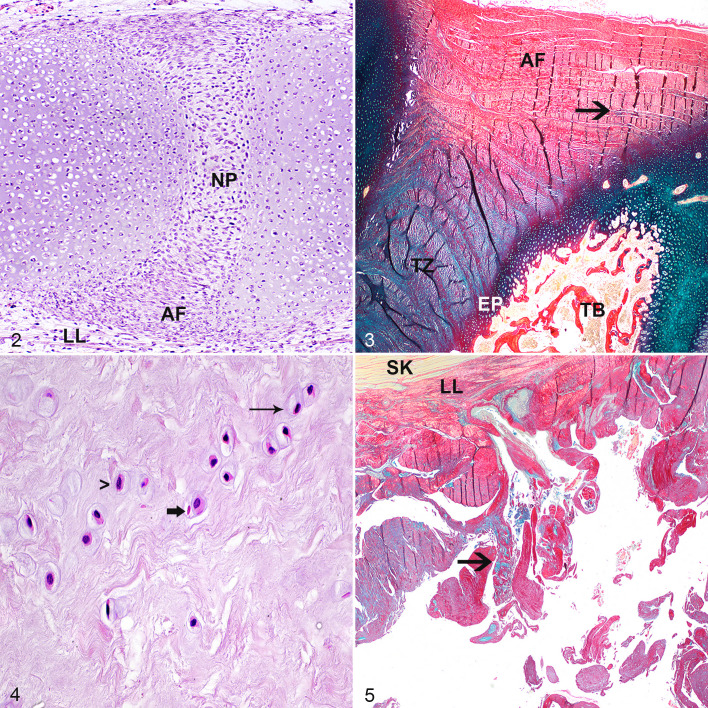
Normal intervertebral disc (IVD), equine fetus of 45 days gestation. The annulus fibrosus (AF) is composed of spindle cells, which form slightly convex rows. The nucleus pulposus (NP) is composed of plumper spindle cells haphazardly embedded in a looser stroma. No cells of notochordal cell phenotype are visible. LL, longitudinal ligament. Hematoxylin and eosin (HE). **Figure 3.** Normal IVD, T1–T2, 6-week-old horse. The AF is composed of ∼10 collagenous lamellae separated by a small amount of mucinous material in some areas (arrow). Alcian blue/picrosirius. TZ, transition zone; EP, end plate; TB, trabecular bone. **Figure 4.** Normal NP, IVD C7–T1, 6-week-old horse. The NP is composed of small spindle to oval chondrocyte-like cells in lacunae within a loose stroma (parameter C, score 1, and parameter E, score 2 [see Table 3]). These cells are either solitary or in rows (thin arrow; parameter D, score 1). Apoptotic chondrocyte-like cells with a pyknotic nucleus and hypereosinophilic cytoplasm are occasionally visible (thick arrow), without the presence of an inflammatory reaction. No tears or clefts are visible (parameter G, score 0). A viable chondrocyte-like cell is shown (>). HE. **Figure 5.** IVD degeneration, C6–C7, horse. Within the severely degenerated AF, the lamellae are torn to the level of the longitudinal ligament (parameter A, score 4) and are interspersed with mucinous material (arrow) with almost complete effacement of the lamellae. SK, skeletal muscle; LL, longitudinal ligament. Alcian blue/picrosirius red.

The fetal AF was composed of spindle cells with a small amount of cytoplasm, which formed about 20 layers of slightly convex rows. The spindle cells of the AF merged dorsally and ventrally into more loosely arranged rows of spindle cells belonging to the longitudinal ligaments (Fig. 2). With Alcian blue/picrosirius red stain, the stroma of the AF was a mixture of blue (mucinous) and slightly red (collagenous) stroma. The fetal NP was composed of more plump spindle cells with a small to moderate amount of eosinophilic cytoplasm, haphazardly embedded in a looser stroma (Fig. 2) that was bright blue in the Alcian blue/picrosirius red stain, compatible with mucinous stroma. No cells with the typical phenotype of notochordal cells as seen in other species (clusters of large cells with cytoplasmic vesicles)^7,46^ were encountered.

In foals from 9 months gestation up to 13 weeks, the AF was composed of fibrous tissue with few small to medium-sized blood vessels in the peripheral two thirds of the AF. With the Alcian blue/picrosirius red stain, the AF appeared to consist of about 10 concave/convex-oriented lamellae of fibroblasts with intercellular collagen fibers (red) and a small (outer layers) to moderate (inner layers) amount of fibrillar pale blue (mucinous) matrix, which increased in amount with age (Fig. 3). From the age of 6 weeks onward, oval cells in lacunae (chondrocyte-like cells) were embedded in this mucinous matrix material, which partly disrupted the lamellae. The NP of foals of 9 months gestation up to 13 weeks was composed of small spindle to oval chondrocyte-like cells in lacunae embedded in a looser stroma. These cells were either solitary, in small groups, or in horizontal or vertical rows (Fig. 4). Rows were most prominent in the cranial and caudal periphery near the cartilaginous end plate. With the Alcian blue/picrosirius red stain, the cytoplasm of these cells stained blue (proteoglycan-rich) and the stroma consisted of a combination of red (collagenous) and blue fibers. The dominant color of these fibers varied by IVD and by location within the NP. Cranially and caudally, the NP was flanked by an often irregular cartilaginous endplate composed of 3 to 6 cell rows of chondroid cells. The trabeculae of the vertebral trabecular bone/subchondral bone flanking the cranial side of the IVD were typically thicker than those of the caudal side with resultant smaller bone marrow spaces.

In 8- to 11-month-old foals, the lamellae of the normal fibroblast-rich AF were increasingly disorganized due to the presence of mucinous extracellular matrix material containing chondrocyte-like cells, with partial effacement of the inner lamellae closest to the NP. In the NP of these foals, more oval than spindle chondrocyte-like cells were present. Occasionally, 2 cells were clustered together. With Alcian blue/picrosirius red stain, the NP stroma was a combination of red and blue fibers without a clear dominant color suggesting an equal distribution of proteoglycans and collagen in the stroma. Increasing numbers of chondrocyte-like cells with a pyknotic nucleus and hypereosinophilic cytoplasm were visible, without the presence of an inflammatory reaction (consistent with apoptosis; Fig. 4).

In 1- to 21-year-old horses, the lamellae of the AF were moderately to extensively disorganized up to the longitudinal ligament, due to the presence of chondrocyte-like cells embedded in mucinous intercellular matrix with almost complete loss of the inner one third of the lamellar architecture. Often the AF contained few to large numbers of tears with well-demarcated yet irregular borders (Fig. 5), and occasionally the AF was completely ruptured.

**Figures 6–9.**
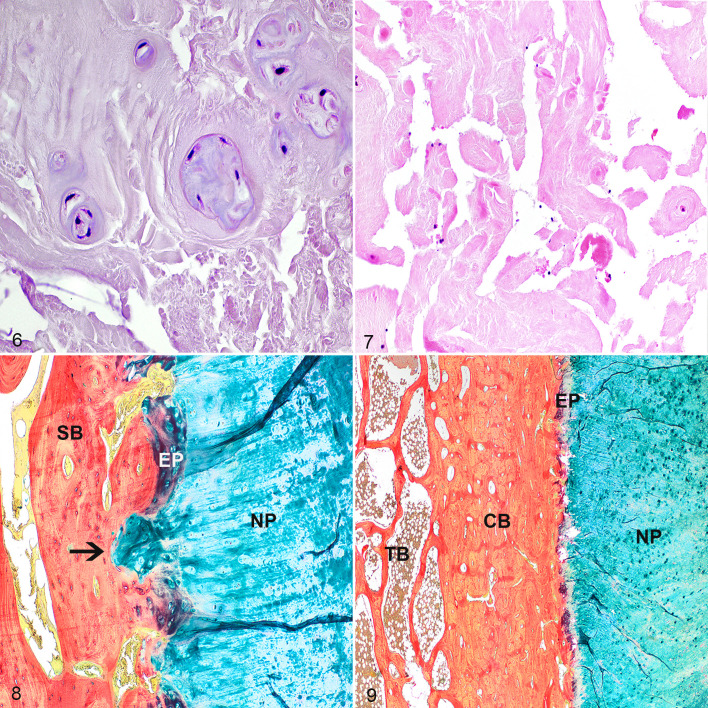
Intervertebral disc (IVD) degeneration, C7–T1, horse. Within the severely degenerated nucleus pulposus (NP), clones of 4 to 6 chondrocyte-like cells form groups (Table 3; parameter C, score 3, parameter D, score 4, parameter E, score 2). The matrix of the NP has intermediate amounts of tears (parameter G, score 2). Hematoxylin and eosin (HE). **Figure 7.** IVD degeneration, C7–T1, horse. The severely degenerated NP is cell-poor and has irregular yet well-demarcated tears (parameter G, score 3). HE. **Figure 8.** IVD degeneration, C6–C7, horse. The end plate (EP) is disrupted with herniation of chondroid NP tissue into the subchondral bone (SB; Schmorl nodule, arrow). Alcian blue/picrosirius red. **Figure 9.** IVD degeneration, C7–T1, horse. The subchondral vertebral bone flanking the cranial site of the IVD is composed of a rim of compact bone (CB) instead of trabecular bone (TB). Alcian blue/picrosirius red.

In these 1- to 21-year-old horses, the NP contained almost exclusively oval (rather than spindle-shaped) chondrocyte-like cells. Within the NP, occasional clones of 3 to 10 chondrocyte-like cells were formed (Fig. 6). Apoptotic chondrocyte-like cells were commonly present, and cell-poor areas became more common with increasing age. With Alcian blue/picrosirius red stain, the stroma of the NP consisted of a combination of red and blue fibers with blue often being the dominant color, suggesting the presence of a more proteoglycan-rich matrix. Infrequently, the NP showed irregular yet well-demarcated tears (Fig. 7). Occasionally, the end plate was disrupted with herniation of NP material into the vertebral subchondral bone (Schmorl nodule; Fig. 8). Also, with increasing age, the vertebral subchondral bone flanking the cranial border of the cartilaginous endplate of the IVD was commonly composed of a rim of compact instead of trabecular bone (Fig. 9). The vertebral bone flanking the caudal border of the IVD also had increasing occurrence of a rim of compact bone, although this started at a later age.

### Validation of the Histological Scoring Scheme

All tissue sections were evaluated and scored histopathologically (Supplemental Table S2) and the percentages and weighted kappa coefficients of the interobserver and intraobserver reliabilities and agreements of the histological variables were determined (Table 1, IVDs of all regions; Table 2, cervical IVDs). Many of the variables showed only limited variation, including morphology of the lamellae of the AF, chondroid metaplasia of the AF, presence of vascular proliferation in the AF, chondroid metaplasia of the NP, cellularity of the NP, presence of notochordal cells in the NP, and subchondral bone sclerosis (Supplemental Table S6).

**Table 1.** Reliability of histologic scores for equine intervertebral disc degeneration: all vertebral regions.^a^

	Intraobserver agreement (%)	Intraobserver reliability (weighted κ)	Interobserver agreement (%)	Interobserver reliability (weighted κ)	Correlation with gross grades 1–5	Correlation with normal versus severe degeneration
AF: morphology	49, 50, 66	0.18, 0.38, 0.53	12, 25	0.12, 0.25	n/a^b^	n/a^b^
AF: chondroid metaplasia	70, 78, 94	0.00, 0.50, 0.53	60, 70	0.00, 0.33	n/a^b^	n/a^b^
AF: tears and cleft formation	51, 56, 62	0.42, 0.62, 0.68	30, 63	0.22, 0.66	n/a^b^	n/a^b^
AF: presence of vascular proliferation	96, 96, 98	0.48, 0.48, 0.85	96, 96	0.44, 0.48	*r* = −0.09; *P* = .56	*r* = −0.03; *P* = .84
NP: chondroid metaplasia	80, 88, 88	0.26, 0.48, 0.59	82, 96	0.36, 0.86	*r* = 0.13; *P* = .36	*r* = 0.24; *P* = .13
NP: chondrocyte-like cell proliferation	54, 60, 74	0.36, 0.62, 0.66	44, 46	0.40, 0.42	*r* = 0.30; *P* = .17	*r* = 0.28; *P* = .08
NP: cellularity	70, 86, 100	n/a^c^, 0.17, 0.48	76, 78	0.00, 0.49	n/a^b^	n/a^b^
NP: presence of notochordal cells	100, 100, 100	n/a^c^	100, 100, 100	n/a^c^	*r* = 0; *P* = 1	*r* = 0; *P* = 1
NP: matrix staining	76, 78, 78	0.69, 0.73, 0.78	62, 74	0.56, 0.73	*r* = 0.20; *P* = .16	*r* = 0.18; *P* = .26
NP: tears and cleft formation	34, 54, 64	0.29, 0.61, 0.73	34, 38	0.36, 0.58	*r* = 0.26; *P* = .07	*r* = 0.36; *P* = 0.02^e^
Endplate morphology	63, 65, 84	0.17, 0.32, 0.34	8, 84	0.02, 0.14	n/a^b^	n/a^b^
Subchondral bone sclerosis	69, 76, 89	0.00. 0.09, 0.50	69, 83	0.00, 0.27	n/a^b^	n/a^b^
Total histological score	n/a^d^	n/a^d^	n/a^d^	n/a^d^	*r* = 0.32; *P* = 0.02^e^	*r* = 0.43; *P* = 0.01^e^

Abbreviations: AF, annulus fibrosis; NP, nucleus pulposus; *r*, Pearson correlation coefficient.

^a^ To evaluate reliability, 50 IVDs were scored twice by 3 independent observers.

^b^ n/a = analysis of the correlation with the gross grade was not applicable because of lack of histological grading reliability.

^c^ n/a = the weighted κ could not be determined because of the high observer agreement.

^d^ n/a = analyses of the observer agreement or weighted κ was not applicable.

^e^ This parameter was significantly correlated with the gross grade of degeneration.

**Table 2.** Reliability of histologic scores for equine intervertebral disc degeneration: cervical vertebral regions.

	Intraobserver agreement (%)	Intraobserver reliability (weighted κ)	Interobserver agreement (%)	Interobserver reliability (weighted κ)	Correlation with gross grades 1–5	Correlation with normal versus severe degeneration
AF: morphology	46, 50, 69	0.22, 0.35, 0.48	41, 45	0.07, 0.13	n/a^a^	n/a^a^
AF: chondroid metaplasia	75, 89, 100	0.00, 0.42, 1	66, 67	0.00, 0.37	n/a^a^	n/a^a^
AF: tears and cleft formation	61, 63, 100	0.53, 0.74, 1	36, 41	0.21, 0.25	*r* = 0.45; *P* = .01^d^	*r* = 0.62; *P* = .001^d^
AF: presence of vascular proliferation	97, 100, 100	0.65, 1, 1	100, 100	1, 1	*r* = 0.13; *P* = .47	*r* = 0.05; *P* = .81
NP: chondroid metaplasia	88, 94, 97	0.00, 0.00, 0.06	91, 97	0.00, 0.04	*r* = 0.12; *P* = .50	*r* = 0.16; *P* = .43
NP: chondrocyte-like cell proliferation	53, 59, 77	0.25, 0.51, 0.61	34, 52	0.13, 0.26	n/a^a^	n/a^a^
NP: cellularity	81, 94, 100	n/a^b^, 0.14, 0.64	78, 94	0.00, 0.00	n/a^a^	n/a^a^
NP: presence of notochordal cells	100, 100, 100	n/a^b^	100, 100, 100	n/a^b^	*r* = 0; *P* = 1	*r* = 0; *P* = 1
NP: matrix staining	69, 69, 81	0.60, 0.65, 0.70	56, 72	0.44, 0.61	*r* = 26; *P* = .15	*r* = 0.19; *P* = .35
NP: tears and cleft formation	38, 59, 66	0.22, 0.63, 0.71	41, 50	0.47, 0.47	*r* = 32; *P* = .07	*r* = 52; *P* = .01^d^
Endplate morphology	58, 77, 88	0.12, 0.16, 0.43	6, 7	0.01, 0.01	n/a^a^	n/a^a^
Subchondral bone sclerosis	67, 72, 80	0.06, 0.18, 0.43	53, 68	0.11, 0.31	n/a^a^	n/a^a^
Total histological score	n/a^c^	n/a^c^	n/a^c^	n/a^c^	*r* = 0.45; *P* = .01^d^	*r* = 0.56; *P* = .003^d^

Abbreviations: AF, annulus fibrosis; NP, nucleus pulposus; *r*, Pearson correlation coefficient.

^a^ n/a = analysis of the correlation with gross degeneration was not applicable because of lack of histological grading reliability.

^b^ n/a = The weighted κ could not be determined because of the high observer agreement.

^c^ n/a = Analyses of the observer agreement or weighted κ was not applicable.

^d^ This parameter was significantly correlated with the gross grade of degeneration.

When including IVDs of all regions in the validation of the scoring scheme, 6 variables could be scored reliably: the presence of vascular proliferation in the AF, chondroid metaplasia of the NP, chondrocyte-like cell proliferation of the NP, presence of notochordal cells, matrix staining of the NP, and tears and cleft formation of the NP. The histological score of these 6 variables combined (ie, the total histological score) was weakly correlated with gross degeneration when taking all gross grades into account, and was moderately correlated when comparing normal IVDs to severely degenerated discs. Of these variables separately, only the presence of tears and clefts in the NP was statistically significantly, but weakly correlated with gross degeneration when comparing normal IVDs (grades 1 and 2) to severely degenerated discs (grades 4 and 5).

The other variables (morphology of the AF, chondroid metaplasia of the AF, tearing of the AF, cellularity of the NP, endplate morphology, subchondral bone sclerosis) could not be scored reliably as revealed by a low percentage for interobserver and/or intraobserver agreement and a low weighted κ interobserver and/or intraobserver agreement coefficient.

When including only the cervical IVDs in the validation of the scoring scheme, tears and clefts of the AF could also be scored reliably but chondrocyte-like cell proliferation of the NP could not. The total score of these 6 histological variables that could be scored reliably in the cervical discs was moderately correlated with gross degeneration.

Of the separate variables, tears and clefts of the AF when including all gross grades, and tears and clefts of both the AF and NP when comparing normal discs to severely degenerated discs, were moderately to strongly correlated with gross degeneration.

These observations taken together formed the basis of a histological scoring scheme for equine IVDs which assesses tears and cleft formation and vascular proliferation of the AF, and chondroid metaplasia, proliferation of chondrocyte-like cells, presence of notochordal cells, matrix staining, and tears and cleft formation of the NP (Table 3).

**Table 3.** Histological scoring scheme for equine intervertebral disc degeneration using both hematoxylin/eosin and Alcian blue/picrosirius red stains.

A. Tears and cleft formation of the annulus fibrosus (only when scoring cervical intervertebral discs)
0 = Absent
1 = Rarely present
2 = Present in intermediate amounts
3 = Abundantly present
4 = Scar/tissue defects
B. Presence of vascular proliferation in the annulus fibrosus
0 = No vascular proliferation
1 = Vascular proliferation
C. Chondroid metaplasia nucleus pulposus
0 = No chondrocyte-like cells, only fibroblast-like cells
1 = Mixture of chondrocyte-like cells and fibroblast-like cells in the inner two thirds of the nucleus pulposus
2 = Presence of chondrocyte-like cells, with formation of rows within in the inner two thirds of the nucleus pulposus
3 = Presence of chondrocyte-like cells, presence of clusters of chondrocyte-like cells
D. Chondrocyte-like cell proliferation of the nucleus pulposus (only when scoring a combination of cervical, thoracic and lumbar intervertebral discs)
1 = Presence of solitary chondrocyte -like cells
2 = Connection of 2 chondrocyte-like cells
3 = Formation of small clones of 3–5 chondrocyte-like cells
4 = Formation of intermediate clones of 6–10 chondrocyte-like cells
5 = Formation of large clones more than 10 chondrocyte-like cells
E. Presence of notochordal cells in the nucleus pulposus
0 = Abundantly present (>50%)
1 = Present (1% to 50%)
2 = Absent
F. Matrix staining of the nucleus pulposus with Alcian blue/picrosirius red staining
0 = Blue (green) stain dominates
1 = Mixture of blue and red staining
2 = Red stain dominates
G. Tears and cleft formation of the nucleus pulposus
0 = Absent
1 = Rarely present
2 = Present in intermediate amounts
3 = Abundantly present
4 = Scar/tissue defects

Furthermore, the total histological score is important to histologically assess degeneration of the equine IVD by histology.

### Immunohistochemistry

No cells in the fetal or adult IVDs were immunolabeled for cytokeratin 18. However, labeling for cytokeratin 18 was present in the cell membrane of notochordal cells (Suppl. Fig. S1) but not the chondrocyte-like cells in the canine control IVD. Renal tubular cells of adult and fetal horses were also immunolabeled for cytokeratin 18 (Suppl. Fig. S2).

### Biochemistry

**Figure 10.**
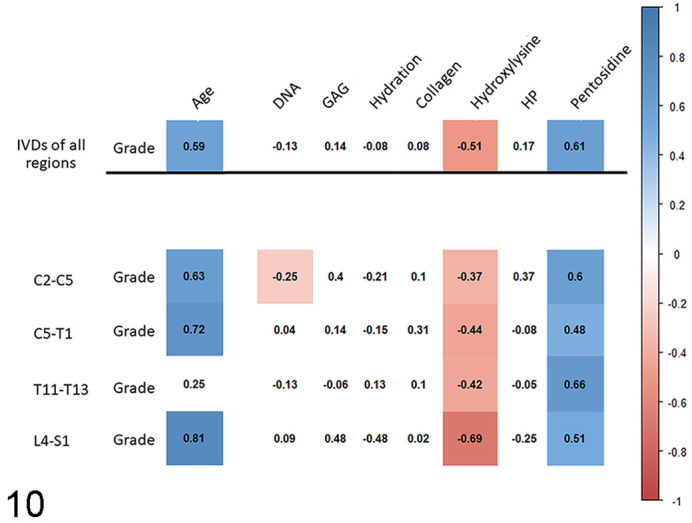
Correlation between degeneration of the nucleus pulposus and biochemical data on equine intervertebral discs. In the heat map, the numbers are Spearman rank correlation coefficients, and the red and blue colored boxes indicate statistically significant (*P* ≤ .05), negative and positive correlations, respectively. IVD, intervertebral disc; C, cervical vertebra; T, thoracic vertebra; L, lumbar vertebra; S, sacral vertebra; DNA, DNA per gram (wet weight); GAG, glycosaminoglycans per gram (wet weight); Collagen, total amount of collagen per gram (wet weight); HP, hydroxyl-lysyl-pyridinoline.

Details on the composition of the normal IVD and the biochemical changes with age and degeneration are depicted in Figures 10 and S9–S14. The most notable, statistically significant biochemical findings are summarized below.

#### Comparison of normal NP versus normal AF

The detailed information on the composition of the normal IVD is depicted in Supplemental Figure S3.

The collagen 1 content of the AF was moderately higher than that of the NP. In the NP the GAG content was slightly higher than in the AF. However, there was no difference in aggrecan or collagen type 2 content and hydration between the AF and the NP. The water content of the AF and NP were similar (AF, 69 ± 12% [mean ± SD]; NP, 70 ± 15%).

**Figures 11–13.**
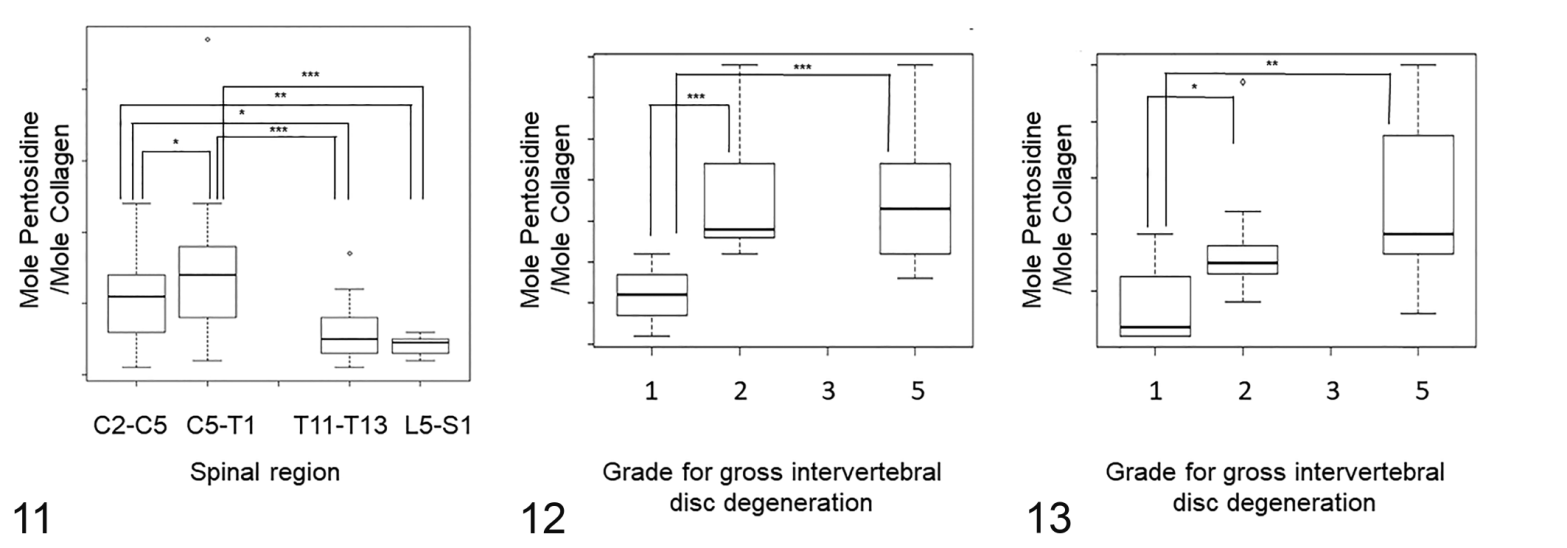
Amount of pentosidine within normal intervertebral discs (IVDs) among the different spinal regions (Fig. 11), and among different gross grades for degeneration in the caudal cervical region (Figs. 12, 13). The box shows the median and first and third quartiles; whiskers show the minimum and maximum; o, outliers. *P < .05, **P < .01, ***P < .001. The amount of pentosidine is expressed as moles of pentosidine per mole of collagen. **Figure 11.** The amount of pentosidine in normal nucleus pulposi (NPs) of the caudal cervical region (C5–T1) was higher than in normal NPs of the cranial cervical region (C2–C5). The amount of pentosidine in normal NPs of the cranial and caudal cervical regions was greater than in normal NPs of the thoracic (T11–T13) and lumbosacral (L4–S1) regions. **Figure 12.** The amount of pentosidine in the annulus fibrosus (AF) of grade 2 and 5 IVDs is greater than in the AF of grade 1 IVDs. **Figure 13.** The amount of pentosidine in the NP of grade 2 and 5 IVDs is greater than in the NP of grade 1 IVDs.

The mean pentosidine content was higher in the caudal cervical NPs than the NPs of the cranial cervical region, and the NPs of both the cranial and the caudal cervical regions had a higher mean pentosidine content than those of the thoracic and the lumbosacral regions (cranial cervical vs caudal cervical: mean 0.32, 95% confidence interval [CI] 0.03–0.61, *P* < .05; thoracic vs cranial cervical: mean 0.30, 95% CI 0.03–0.57, *P* < .05; thoracic vs caudal cervical: mean 0.62, 95% CI 0.27–0.97, *P* < .001; lumbosacral vs cranial cervical: mean 0.45, 95% CI 0.14–0.76, *P* < .01; lumbosacral vs caudal cervical: mean 0.76, 95%CI 0.37–1.16, *P* < .001; Fig. 11).

For the normal AFs, there was no statistically significant difference in the mean pentosidine content between the different regions.

#### Association with age

Detailed information can be found in the Supplemental Figures S4 and S5.

A strong to very strong increase in pentosidine (with exception of the caudal cervical NPs) and a moderate to very strong decrease of hydroxylysine was noted in both the AF and the NP. Furthermore, there was a strong increase in total collagen in the caudal cervical NPs. In contrast to expectations, the GAG content of the caudal cervical NPs increased very strongly with age.

#### Association with degeneration

Detailed information about the biochemical changes associated with gross degeneration can be found in Figures 10 and Supplemental Figures S6–S8.

The most important findings were a moderate to strong increase in pentosidine in the AF and the NP, and a moderate (AF) and weak to severe (NP) decrease of hydroxylysine.

In the linear mixed model, the mean pentosidine content in the caudal cervical AFs and NPs of both grade 2 and grade 5 IVDs was higher than those of grade 1 IVDs (AF grade 1 vs 2: mean 1.40, 95% CI 0.68–2.10, *P* < .001; AF grade 1 vs 5: mean 1.40, 95% CI 0.73–2.06, *P* < .001; NP grade 1 vs 2: mean 1.25, *P* < .05; NP grade 1 vs 5: mean 1.49, *P* < .01; Figs. 12, 13).

Furthermore, the collagen type 1 content of the NP increased weakly with degeneration. In contrast to expectations, there was no change in GAG content with degeneration in the NP.

## Discussion

The morphology of the equine IVD and its gross changes in degeneration are largely similar to those in dogs and humans.^10^ However, the present study demonstrated that aspects of the cellular and biochemical composition, biochemical changes associated with age, and the histological and biochemical changes associated with degeneration differ in horses from those reported in dogs and humans.

Although 6 out of 12 histological variables could be scored reliably, only the total histological score, tearing of the NP, and tearing of the cervical AF correlated with gross degeneration. Thus, these variables can be used to histologically distinguish normal from degenerated discs, especially for the cervical discs. Tearing is an important characteristic in gross grading^10^ and can also be seen in degenerated equine discs on magnetic resonance imaging^60^ and therefore it is not surprising that this variable is also of use in histological scoring. Consequently, gross grading is considered to be the leading method for assessment of IVDD in horses.

The histological scoring scheme previously validated for canine disc degeneration^7^ was modified to include vascular proliferation in the outer AF because this is a phenomenon that has not been described in dogs and could represent tissue repair induced by microtrauma of the outer AF in horses, although a correlation with gross degenerative changes was not found. It was assumed that degeneration of the IVD starts in the NP, as in dogs and humans; therefore, chondroid metaplasia of the NP was included in the proposed scoring scheme despite the fact that the present study did not show a reliable association of this variable with gross IVD degeneration.

Surprisingly, notochordal cells were not seen in any of the IVDs in the current study, although they have an important role in organogenesis of the vertebral column. In children and in the normal discs of adult non-chondrodystrophic dogs, notochordal cells are present in the NP and are replaced by smaller, nonvacuolated chondrocyte-like cells during maturation in humans and during degeneration in the dog.^12,26^ In mammals, including the horse, the NP is assumed to be formed by the notochord, which itself disappears during late embryogenesis.^5,54^ However, the presence of cells with morphological characteristics of notochordal cells could not be confirmed during gestation or postnatally. This finding was substantiated by using immunohistochemistry for cytokeratin 18, a marker expressed by notochordal cells.^45^ According to a previous publication, the formation of the equine NP occurs after gestation day 30, although the exact day of the formation of the NP is not mentioned.^5^ In vitro and in vivo studies in juvenile and adult mice, adult rabbits, and juvenile pigs have shown that notochordal cells can transform into chondrocyte-like cells, implying that these morphologies are not different cell linages but instead represent different stages of cell activation, differentiation, or metaplasia.^13,31,42,44,65^ Although unlikely, it can therefore not be ruled out that the equine NP is initially formed from the notochord with transformation of the notochordal cells into chondrocyte-like cells between gestation days 30 and 45, the age of the youngest fetus. Still, it can be concluded that the absence of cells morphologically consistent with physaliferous notochordal cells is not associated with maturation or degeneration of the NP in the horse.

The most important biochemical changes associated with age and degeneration of both the AF and the NP of the equine IVD was a reduction in hydroxylysine and an increase in pentosidine content. Hydroxylysine has 2 important functions in the synthesis of collagen. It plays an important role in collagen cross-linking and in posttranslational enzymatic O-glycosylation. Hydroxylysine is furthermore implicated in the formation of nonenzymatic AGEs.^14,53,56,63^ The decrease of hydroxylysine does not appear to affect crosslinking because the amount of HP, one of the crosslinks formed via hydroxylysine, was not negatively associated with age nor degeneration. It is likely that the reduced hydroxylysine content with age and IVDD is for a large part the result of formation of AGEs, as the decline in hydroxylysine and the increase in AGEs seems to go hand in hand. Hydroxylysine-linked glycosylation is acid-labile and could therefore not be determined after hydrolyzation with hydrochloric acid. However, the decrease in hydroxylysine with age and IVDD may also indicate decreased glycosylation.^64^ Although the exact function of glycosylation of hydroxylysine is yet unknown, it does appear to play a role in collagen crosslink maturation and therefore it likely has an effect on the biological function of collagen.^14,53,56^ Hence, the data may suggest that a decrease in glycosylation of hydroxylysine affects the biomechanical properties of affected IVDs.

Similar to the equine IVD, an increase in the amount of pentosidine with age and degeneration has been described in human IVDs.^1,40,61^ Pentosidine is the best-characterized AGE crosslink^48^ and is often used as biomarker for nonenzymatic glycation^47^ because it is metabolically stable and correlates well with tissue levels of other AGEs.^48^ An increase in AGEs has been shown to be responsible for yellow discoloration, loss of compliance, increase in tissue stiffness, loss of strength, brittleness, and loss of viscoelasticity of the affected tissues including the IVD.^1,3,20,22,61^ The increase in pentosidine content and its biomechanical consequences can therefore explain the typical gross changes of tearing and yellow discoloration seen in equine IVDD.

The IVDs with a normal gross morphology in the caudal cervical spine had statistically significantly higher pentosidine levels in the NP compared to the other regions. Considering that also gross degeneration is most prevalent in this region,^10^ this indicates accelerated degeneration in the caudal cervical region and in addition suggests that IVDD starts in the NP, as in other species.^36^ In addition to age,^3,14,20^ oxidative stress contributes to the formation of AGEs.^21,36^

Within an individual, purely age-related degeneration is expected to be the same in all IVDs, and more severe degeneration in a specific region can occur due to increased mechanical loading in that area.^1,25^ Chronic abnormal loading can cause adverse changes in the composition of the IVD’s extracellular matrix,^15,25^ and it is feasible that increased mechanical loading also leads to microtrauma with resultant production of reactive oxygen species and glycoxidation causing accumulation of AGEs.^36^ Therefore, increased mechanical loading of the caudal cervical region could be an explanation for the differences in AGE content in this region compared to the other spinal regions.

In vitro studies have shown that accumulation of AGEs in the human NP can cause a decrease in the production of aggrecan and initiate inflammation-related degeneration via binding to the receptor RAGE (receptor for advanced glycation end-products), and in this way AGEs can contribute to the initiation and progression of IVDD.^50,67^ However, in the current study, aggrecan levels were not correlated with degree of degeneration, and thus decrease in production of aggrecan due to AGEs does not seem to be important in the equine disc. Whether or not AGEs initiate inflammation in the equine IVD needs to be further investigated, but a classical inflammatory reaction has not been seen histologically, nor is there substantial neovascularization of the entire IVD.

In contrast to the common NP- and AF-specific biochemical profiles described in dogs and humans, the equine NP of normal IVDs (of all regions combined) only contained slightly more GAGs than the AF, and there was no statistically significant difference in hydration (both were ∼70%). On the contrary, the canine AF contains ∼60% and the NP ∼81% water and the human AF contains ∼66% to 70% and the NP ∼80% to 89% water.^2,27,58^ This similar water content in NP and AF of horses may explain why the NP in the normal equine IVD cannot be easily distinguished macroscopically from the AF, in contrast to the IVD of dogs and humans.^10^

Furthermore, the aggrecan and GAG content in the equine disc, which strongly correlates with the amount of hydration, did not significantly decrease with age or degeneration as is described in dogs and humans.^1,2,7,8^ Loss of GAGs and subsequently of water content are important factors in collapse of severely degenerated discs in dogs and humans^1,6^ and the constancy of hydration of the equine IVD between the different grades for degeneration could explain why loss in disc height is not seen in equine IVDD.^10^ In fact, the GAG content increased in the equine AF and NP with age and in the AF with degeneration.

Furthermore, histologically, GAG-rich mucinous stroma with associated chondrocyte-like cells were commonly seen in the AFs. Proliferation of chondrocyte-like cells and subsequent disorganization of the lamellar structure of the AF are reliable histological indicators of degeneration in dogs and humans.^7,46^ In the horse, however, disorganization of lamellar architecture and chondroid metaplasia of the AF could not be scored reliably, yet moderate to marked disorganization and rupture of the lamellae and marked chondroid metaplasia were seen in the vast majority of the scored IVDs. Therefore, lamellar disorganization due to chondroid metaplasia is probably a normal feature of the equine IVD. The presence of chondroid material in the AF of the equine IVD has been described before in a study of 255 cervical IVDs from 17 horses.^68^ In that study, chondroid material in the AF was only seen in 5 IVDs and interpreted as a Hansen type 2 herniation. However, only hematoxylin/eosin stains were used to evaluate the histological characteristics of the equine IVD, which could explain why disorganization of the AF by chondroid metaplasia was not clearly visible in the other IVDs examined, since Alcian blue/picrosirius red stain is more suitable to highlight the chondroid intercellular matrix than hematoxylin/eosin.^23^

Both aged and degenerated NPs showed a slight fibrotic phenotype of the extracellular matrix in the biochemical assays. Interestingly, the fibrotic phenotype with age was predominantly seen in the caudal cervical discs, contributing to the suspicion of distinctive biomechanical stress in this specific vertebral region.^10,49^ Furthermore, the fact that with degeneration an increased fibrotic phenotype is chiefly present in the NP further supports the suggestion that degeneration starts in the NP. However, the fibrotic phenotype of the extracellular matrix in the degenerated equine IVD was mild, and Alcian blue/picrosirius staining of the matrix was not correlated with degeneration even though this variable could be scored reliably. This is in contrast to dogs and humans.^7,46^ Still, it seems feasible that this weak fibrotic phenotype could impair the physiological function of the NP and contribute to changes in biomechanical properties such as motion stiffness and loss of elasticity of the degenerated equine IVD.^34^

In conclusion, tearing of the NP and the cervical AF could be reliably scored histologically and were correlated with degeneration. The total histological score included tears and cleft formation and vascular proliferation of the AF, and chondroid metaplasia, proliferation of chondrocyte-like cells, presence of notochordal cells, matrix staining, and tears and cleft formation of the NP, and this total histological score also correlated with degeneration. Therefore, these histological variables can be of use to distinguish normal from degenerated equine IVDs, although it did not offer advantages over gross grading. Biochemically, ageing and degeneration of the equine IVD were characterized by changes in the composition of the NP and less so of the AF, consisting of a significant increase of AGEs and a mild fibrotic biochemical phenotype of the extracellular matrix. These biochemical changes were most prominent in the caudal cervical region. They can explain the typical gross changes of yellow discoloration and cleft formation, and they likely result in altered biomechanical properties that can subsequently cause degenerative changes of spatially close structures such as the articular process joints. As such, we speculate that equine IVDD might contribute to cervical neurological signs as seen in cervical vertebral myelopathy and this should be an area of further study. A decrease in GAGs and subsequent dehydration, the most important biochemical alteration in canine and human IVDD, were not seen in the horse. Furthermore, notochordal cells could not be identified in the equine NP, not even in fetal IVDs.

## Supplemental Material

Supplemental Material, sj-pdf-1-vet-10.1177_03009858211067463 - Intervertebral disc degeneration in warmblood horses: Histological and biochemical characterization Click here for additional data file. Supplemental Material, sj-pdf-1-vet-10.1177_03009858211067463 for Intervertebral disc degeneration in warmblood horses: Histological and biochemical characterization by Wilhelmina Bergmann, Chris van de Lest, Saskia Plomp, Johannes C. M. Vernooij, Inge D. Wijnberg, Willem Back, Andrea Gröne, Mark W. Delany, Nermin Caliskan, Marianna A. Tryfonidou and Guy C. M. Grinwis in Veterinary Pathology

Supplemental Material, sj-xlsx-1-vet-10.1177_03009858211067463 - Intervertebral disc degeneration in warmblood horses: Histological and biochemical characterization Click here for additional data file. Supplemental Material, sj-xlsx-1-vet-10.1177_03009858211067463 for Intervertebral disc degeneration in warmblood horses: Histological and biochemical characterization by Wilhelmina Bergmann, Chris van de Lest, Saskia Plomp, Johannes C. M. Vernooij, Inge D. Wijnberg, Willem Back, Andrea Gröne, Mark W. Delany, Nermin Caliskan, Marianna A. Tryfonidou and Guy C. M. Grinwis in Veterinary Pathology
