# Supplementary material for: Intervertebral disc degeneration in warmblood horses: Histological and biochemical characterization
Source: Vet Pathol. 2022 Jan 4;59(2):284–98. doi: 10.1177/03009858211067463 (PMC8928235; doi:10.1177/03009858211067463)
Supplement: Supplemental Material, sj-pdf-1-vet-10.1177_03009858211067463 - Intervertebral disc degeneration in warmblood horses: Histological and biochemical characterization [file sj-pdf-1-vet-10.1177_03009858211067463.pdf]

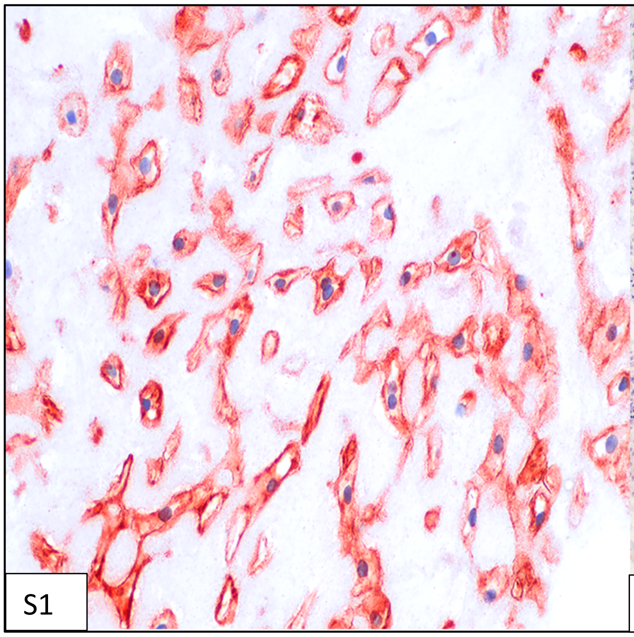
S1

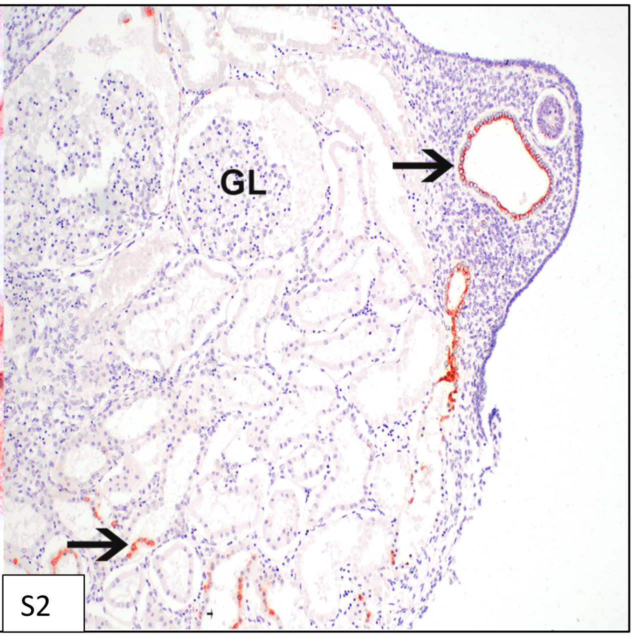

S2

Supplemental Figure S1. Nucleus pulposus of a herniated intervertebral disc, non-chondrodystrophic dog. In the nucleus pulposus are cytokeratin 18 positive notochordal cells. IHC for Cytokeratin 18.

Supplemental Figure S2. Normal kidney, horse, fetus of 45 days gestation. The distal and collecting tubules are cytokeratin 18 positive (arrows). GL= glomerulus. IHC for Cytokeratin 18.

S3

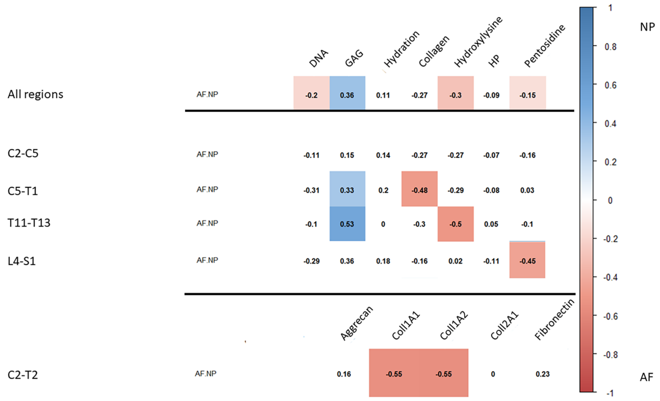

Supplemental Figure S3. Heat maps of Spearman rank correlation coefficients of the composition of the intervertebral disc (IVD). A red color is given when the amount of the variable is statistically significantly higher in the annulus fibrosus compared to the nucleus pulposus. A blue color is given when the amount of the variable is statistically significantly higher in the nucleus pulposus compared to the annulus fibrosus. $P \leq 0.05$. C= cervical vertebra. T= thoracic vertebra. L=lumbar vertebra. S=sacral vertebra. DNA= DNA per gram wet weight. GAG= glycosaminoglycans per gram wet weight. collagen= total amount of collagen per gram wet weight. HP= hydroxyl-lysyl-pyridinoline.

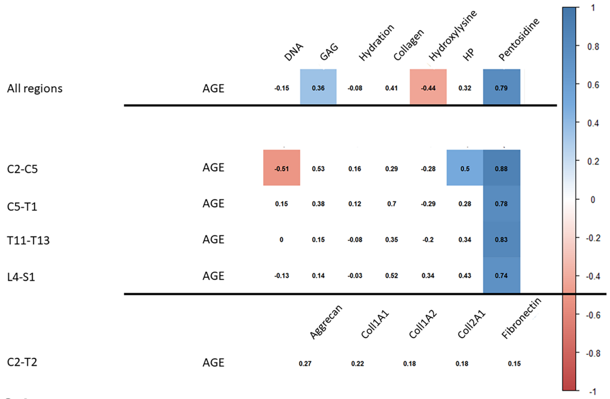

S4

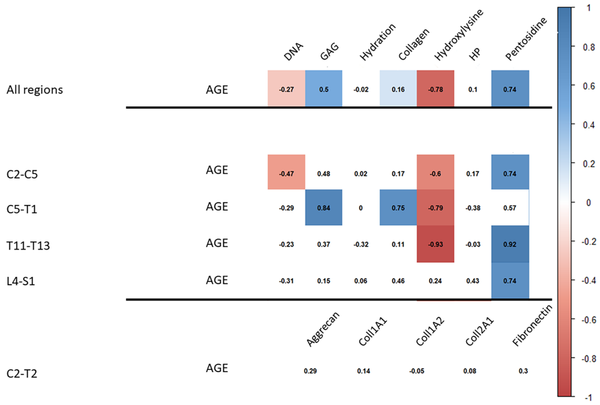

S5

Supplemental Figures S4 and S5. Heat maps of Spearman rank correlation coefficients of age related changes of the intervertebral disc (IVD). A red color is given when there is a negative statistically significant correlation and a blue color is given when there is a positive statistically significant correlation. P ≤ 0.05.
C= cervical vertebra. T= thoracic vertebra. L=lumbar vertebra. S-sacral vertebra. Normdeg= normal versus severely degenerated. DNA= DNA per gram wet weight. GAG= glycosaminoglycans per gram wet weight. collagen= total amount of collagen per gram wet weight. HP= hydroxyl-lysyl-pyridinoline. **Figure S4.** Age related changes of the annulus fibrosus. **Figure S5.** Age related changes of the nucleus pulposus.

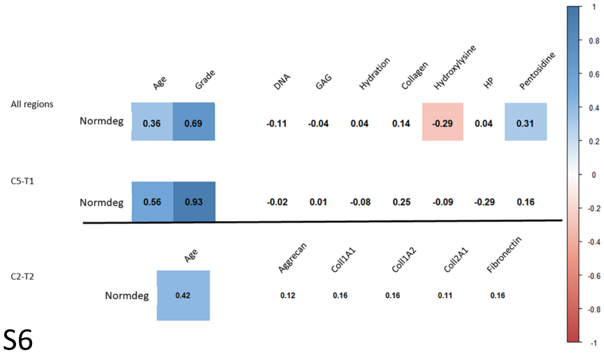

S6

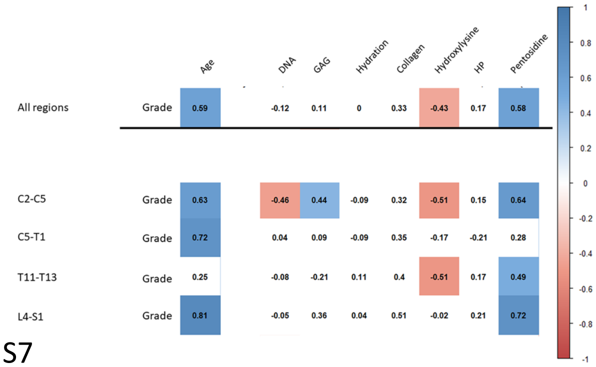

S7

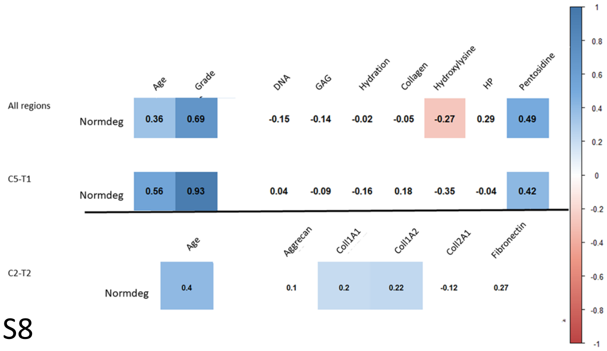

S8

Supplemental Figures S6-S8. Heat maps of Spearman rank correlation coefficients of degeneration related changes of the intervertebral disc (IVD). A red color is given when there is a negative statistically significant correlation and a blue color is given when there is a positive statistically significant correlation. P ≤ 0.05.
C= cervical vertebra. T= thoracic vertebra. L=lumbar vertebra. S-sacral vertebra. Normdeg= normal versus severely degenerated DNA= DNA per gram wet weight. GAG= glycosaminoglycans per gram wet weight. collagen= total amount of collagen per gram wet weight. HP= hydroxyl-lysyl-pyridinoline.
Figure S12. Degeneration related changes of the annulus fibrosus of the normal versus the severely degenerated intervertebral disc.
Figure S13. Degeneration related changes of the annulus fibrosus.
Figure S14. Degeneration related changes of the nucleus pulposus of the normal versus the severely degenerated intervertebral disc.

Supplemental Table S1. Signalment and experimental usage of the intervertebral discs of the warmblood horses in this study
The sampled area and the amount of discs used are noted in brackets.

case number	breed	gender	cause of death	age	gross scoring	histological evaluation	biochemical evaluation
1	RDSH	mare	death dam	45 days gestation	no	yes (whole vertebral column, 29)	no
2	RDSH	unknown	aborted as part of a study	61 days gestation	no	yes (whole vertebral column, 29)	no
3	RDSH	mare	torsio umbilicalis	9 months gestation	no	yes (C3-T2, 6)	no
4	RDSH	mare	necrotizing placentitis	9 months gestation	no	yes (T11-T12, 2; L5-S1, 2)	no
5	RDSH	unknown	interstitial pneumonia and necrotizing hepatitis	10 months gestation	no	yes (C3-C4, 1; C6-C7, 1)	no
6	RDSH	mare	herpes viral hepatitis	0 day	no	yes (C3-C4, 1; C6-C7, 1)	no
7	RDSH	mare	herpes viral pneumonia and hepatitis	1 day	no	yes (C3-C4, 1; C6-C7, 1)	no
8	RDSH	unknown	interstitial pneumonia	4 days	no	yes (C3-C4, 1; C6-C7, 1)	no
9	RDSH	stallion	clostridial enterocolitis	5 days	no	yes (C3-C4, 1; C6-C7, 1)	no
10	RDSH	mare	clostridial enteritis	5 days	no	yes (C3-C4, 1; C6-C7, 1)	no
11	RDSH	mare	fibrinosuppurative typhlocolitis and purulent hepatitis	7 days	no	yes (C3-C4, 1; C6-C7, 1)	no
12	RDSH	stallion	herpes viral pneumonia	8 days	no	yes (C3-C4, 1; C6-C7, 1)	no

13	RDSH	stallion	interstitial pneumonia	10 days	no	yes (C3-C4, 1; C6-C7, 1)	no
14	Zangersheide	stallion	hydrocephalus	6 weeks	no	yes (C4-T2, 5)	no
15	RDSH	stallion	necrotizing colitis	13 weeks	no	yes (C3-C4, 1; C6-C7, 1)	no
16	RDSH	stallion	anesthesia death after dental surgery	8 months	yes	Yes (C2-C3, 1; C7-T2, 2; T11-T12, 2; L5-S1, 2).	yes (C2_C3,1; C6-C7, 1; T11-T13, 2; L5-S1,2)
17	RDSH	mare	fungal eustachitis	11 months	yes	Yes (C2-T2, 7; T12-T12, 1)	yes (C2-C3, 1; C6-C7, 1; T11-T13, 2)
18	Zangersheide	stallion	invagination caecum	1 year	yes	no	yes (C3-C4,1; C6-C7, 1; T11-T13, 2)
19	RDSH	stallion	cervical ataxia	2 years	yes	yes (C5-C7, 2; T11-T12, 1; L6-S1, 1)	no
20	Holsteiner	gelding	cervical and back pain	4 years	yes	yes (C2C3, 1; C7T1, 1; L6S1, 1)	yes (C2-C3, 1; C6-C7, 1; T11-T13, 2)
21	RDSH	mare	cervical ataxia	5 years	yes	yes (C2-C3, 1; C6-T1, 2; L6-S1, 1)	no
22	RDSH	gelding	kissing spines	6 years	yes	yes (C2-C3, 1; T11-T12, 1; L5-S1, 2)	yes (C2-C3, 1; L5-S1, 2)

23	RDSH	mare	suspensory desmitis	7 years	yes	no	yes (C2-C3, 1; C6-C7, 1; T11-T13, 2; L5-L6, 1)
24	RDSH	gelding	myopathy	7 years	yes	yes (C2-T1, 6)	no
25	RDSH	mare	dislocation cervical vertebra	10 years	yes	yes (C3-T1, 5; T11-T12, 1; L5-S1, 2))	no
26	RDSH	gelding	cervical ataxia	11 years	yes	yes (C2-T2, 7)	no
27	RDSH	gelding	cervical ataxia	11 years	yes	yes (C3-C5, 2; C7-T1, 1; L5-S1, 2)	yes (C2-C3, 1; C6-T1, 2; T11-T13, 2; L5-S1,2)
28	RDSH	gelding	equine multinodular pulmonary fibrosis	12 years	yes	yes (C3-C4, 1; T12-T13, 1; L6-S1, 1)	no
29	RDSH	gelding	cervical ataxia	12 years	yes	yes (C3-T1, 5; T11-T12, 2; L5-S1, 2)	no
30	RDSH	mare	colic	13 years	yes	no	yes (C2-T1, 6)
31	RDSH	mare	displacement colon	13 years	yes	no	yes (C2-T2, 7)
32	RDSH	mare	fibrinous peritonitis	15 years	yes	yes (C6-C7, 1; T11-T12, 2)	no
33	RDSH	mare	gallstones	15 years	yes	yes (C3-C4, 1; C6-C7, 1; L5-S1, 2)	no
34	RDSH	gelding	cervical ataxia	16 years	yes	yes (T11-T12, 2)	no

35	RDSH	mare	torsion colon	16 years	yes	yes (C4-T1, 4; T12-T13, 1; L5-S1, 2)	yes (C6-C7,1; T11-T13, 2)
36	RDSH	mare	colic	17 years	yes	no	yes (C2-C5, 3; C6-T1, 2; T11-T13, 2; L5-S1, 2)
37	RDSH	gelding	foramen epiploicum strangulation	18 years	yes	yes (C2-C7, 5; T12-T13, 1; L6-S1, 1)	yes (C6C7, 1; T11-T13, 2; L5L6, 1)
38	RDSH	mare	neuroma	18 years	yes	yes (C2-C7 , 5; T11-T13, 2)	no
39	RDSH	mare	arthrosis	18 years	yes	yes (C2-C6, 4; C7-T1,1)	no
40	RDSH	gelding	hemothorax	21 years	yes	yes (C3-C4, 1)	no
41	RDSH	gelding	maxillary carcinoid	21 years	yes	no	yes (C2-T2, 7; T11-T13, 2; L5-S1, 2)

RDSH = Royal Dutch Sport Horse
C= cervical vertebra, T= Thoracic vertebra, L= lumbar vertebra, S= sacral vertebra

Veterinary Pathology: Supplemental Materials

Bergmann et al. Intervertebral disc degeneration in warmblood horses: histological and biochemical characterization.

Supplemental Table S2. Histological scoring scheme for equine intervertebral disc degeneration using both hematoxylin/eosin and Alcian blue/picrosirius red stains.

A. Morphology of the lamellae of the annulus fibrosus (best viewed in the Alcian blue/picrosirius red stain; area with the highest score)

0 Well-organized, half ring-shaped, collagen lamellae
1 Mild disorganized; some loss of half ring-shaped structure, most lamellar layer, still distinguishable (<25%)
2 Moderately disorganized; partly ruptured annulus fibrosus, loss of half ring-shaped structure (25-75%)
3 Completely ruptured annulus fibrosus; no or few distinguishable half ring-shapedcollagen lamellae (>75%)

0

B. Chondroid metaplasia of the annulus fibrosus

0 No chondrocyte morphology, just spindle-shaped cells
1 Mild chondrocyte-like cell proliferation (i.e. limited to inner most annulus fibrosus layers) 4
2 Moderate chondrocyte-like cell proliferation (i.e. chondrocyte-like cells cells in up to half ofthe annulus fibrosus)
3 Marked chondrocyte-like cell proliferation (i.e. chondrocyte-like cells up to outer layersof the annulus fibrosus)

C. Tears and cleft formation of the annulus fibrosus

0 Absent
1 Rarely present
2 Present in intermediate amounts
3 Abundantly present
4 Scar/tissue defects

D. Presence of vascular proliferation in the annulus fibrosus

0 No vascular proliferation
1 Vascular proliferation

E. Chondroid metaplasia nucleus pulposus

0 No chondrocyte-like cells, only spindle cells
1 Mixture of chondrocyte-like cells and spindle cells in the inner 2/3 of the nucleus pulposus
2 Presence of chondrocyte-like cells, with formation of rows within in the inner 2/3 of the nucleus pulposus
3 Presence of chondrocyte-like cells, presence of clusters of chondrocyte-like cells

F. Chondrocyte-like cell proliferation of the nucleus pulposus

1 Presence of solitary chondrocyte-like cells
2 Connection of two chondrocyte-like cells
3 Formation of small clones of 3-5 chondrocyte-like cells
4 Formation of intermediate clones of 6-10 chondrocyte-like cells
5 Formation of large clones more than 10 chondrocyte-like cells

G. Cellularity of the nucleus pulposus taken from the most cell poor area

0 No areas without viable cells present per high power field (400 x)
1 > 75 % of space of a high power field (400 x) is occupied by cells
2 50-75 % of space of a high power field (400x) is occupied by cells
3 25-50 % of space of a high power field (400x) is occupied by cells
4 < 25 % of space of a high power field (400x) is occupied by cells

H. Presence of notochordal cells in the nucleus pulposus

0 Abundantly present (>50%)
1 Present (1–50%)
2 Absent

I. Matrix staining of the nucleus pulposus with Alcian blue/Picrosirius red staining

0 Blue (green) stain dominates
1 Mixture of blue and red staining
2 Red stain dominates

J. **Tears and cleft formation of the nucleus pulposus**

0 Absent
1 Rarely present
2 Present in intermediate amounts
3 Abundantly present
4 Scar/tissue defects

K. Endplate morphology

0 Regular thickness; homogeneous structure
1 Slightly irregular thickness
2 Moderately irregular thickness
3 Severely irregular thickness with interruption of the endplate

L. Subchondral bone sclerosis

0 No sclerosis
1 Cranial formation of compact bone
2 Cranial and caudal formation of compact bone

Supplemental Table S3. Grades for intervertebral disc degeneration used for biochemical analysis distributed among the different spinal regions.[a]

Region	Total	Grade 1	Grade 2	Grade 3	Grade 4-5
Cranial cervical	19	4	13	2	0
Caudal cervical	19	5	5	0	9
Cranial thoracic	2	1	0	0	1
Thoracic	20	7	8	5	0
Lumbo-sacral	13	5	4	4	0

[a] Number of discs used for biochemical analysis.

Supplemental Tables S4 and S5 are posted separately.

Supplemental Table S6. Histological variables with limited variation.

Histological variable	Most common scores given	
	all regions	cervical
AF: Morphology of the lamellae	35 % moderate, 55% complete	37 % moderate, 53 % complete
AF: Chondroid metaplasia	80 % marked	82 % marked
AF Presence of vascular proliferation	95 % positive presence	96 % positive presence
NP: Chondroid metaplasia	presence of clusters in 82 %	presence of clusters in 92 %
NP: Cellularity	most cell poor in 82 %	most cell poor in 95 %
NP: Presence of notochordal cells	100 % no notochordal cells	100 % no notochordal cells
Subchondral bone sclerosis	cranial and caudal compact bone in 76 %	cranial and caudal compact bone in 73 %
